# A novel patient-specific three-dimensional-printed external template to guide iliosacral screw insertion: a retrospective study

**DOI:** 10.1186/s12891-018-2320-3

**Published:** 2018-11-13

**Authors:** Fan Yang, Sheng Yao, Kai-fang Chen, Feng-zhao Zhu, Ze-kang Xiong, Yan-hui Ji, Ting-fang Sun, Xiao-dong Guo

**Affiliations:** Department of Orthopaedics, Union Hospital, Tongji Medical College, Huazhong University of Science and Technology, Wuhan, 430022 China

**Keywords:** External template, Iliosacral screw, Novel navigation tool, Pelvic fracture, Minimal invasive

## Abstract

**Background:**

Iliosacral screw fixation is a popular method for the management of posterior pelvic ring fractures or dislocations, providing adequate biomechanical stability. Our aim in this study was to describe the use of a new patient-specific external template to guide the insertion of iliosacral screws and to evaluate the efficacy and safety of this technique compared with the conventional fluoroscopy-guided technique.

**Methods:**

This was a retrospective study of patients with incomplete or complete posterior pelvic ring disruptions who required iliosacral screw fixation. For analysis, patients were divided into two groups: the external template group (37 screws in 22 patients) and the conventional group (28 screws in 18 patients). The operative time per screw, radiation exposure time and the rate of screw perforation (accuracy) were compared between groups. In the external template group, the difference between the actual and planned iliosacral screw position was also compared.

**Results:**

In the conventional group, the average operative time per screw was 39.7 ± 10.6 min, with an average radiation exposure dose of 1904.0 ± 844.5 cGy/cm^2^, with 4 cases of screw perforation. In the external template group, the average operative time per screw was 17.9 ± 4.7 min, with an average radiation exposure dose of 742.8 ± 230.6 cGy/cm^2^ and 1 case of screw perforation. In the template group, the mean deviation distance between the actual and planned screw position was 2.75 ± 1.0 mm at the tip, 1.83 ± 0.67 mm in the nerve root tunnel zone and 1.52 ± 0.48 mm at the entry point, with a mean deviation angle of 1.73 ± 0.80°.

**Conclusions:**

The external template provides an accurate and safe navigation tool for percutaneous iliosacral screw insertion that could decrease the operative time and radiation exposure.

## Background

Iliosacral (IS) screw fixation is a popular method for the management of posterior pelvic ring fractures or dislocations, providing adequate biomechanical stability [1]. The classic percutaneous technique, as described by Matta and Saucedo [2], uses fluoroscopic guidance to reduce the extent of soft tissue disruption, decrease the volume of blood loss and shorten the overall operative time. However, fluoroscopic guidance is a significant source of radiation exposure to both the patient and medical staff. Additionally, patient-specific factors, such as obesity, intestinal gas and a dysmorphic sacrum, have been associated with a screw malposition rate of 2–15%, which may lead to catastrophic neurovascular injury [3, 4].

The use of computer-assisted navigation systems has been advocated to lower the risk of screw malposition, and of associated neurovascular injury, as well as to decrease exposure to radiation during IS screw insertion [5–7]. Although these systems do provide greater accuracy and less radiation exposure, their high cost and the complexity of the setup have limited the widespread application of these systems in intermediate- and primary-care hospitals.

**Fig. 1:**
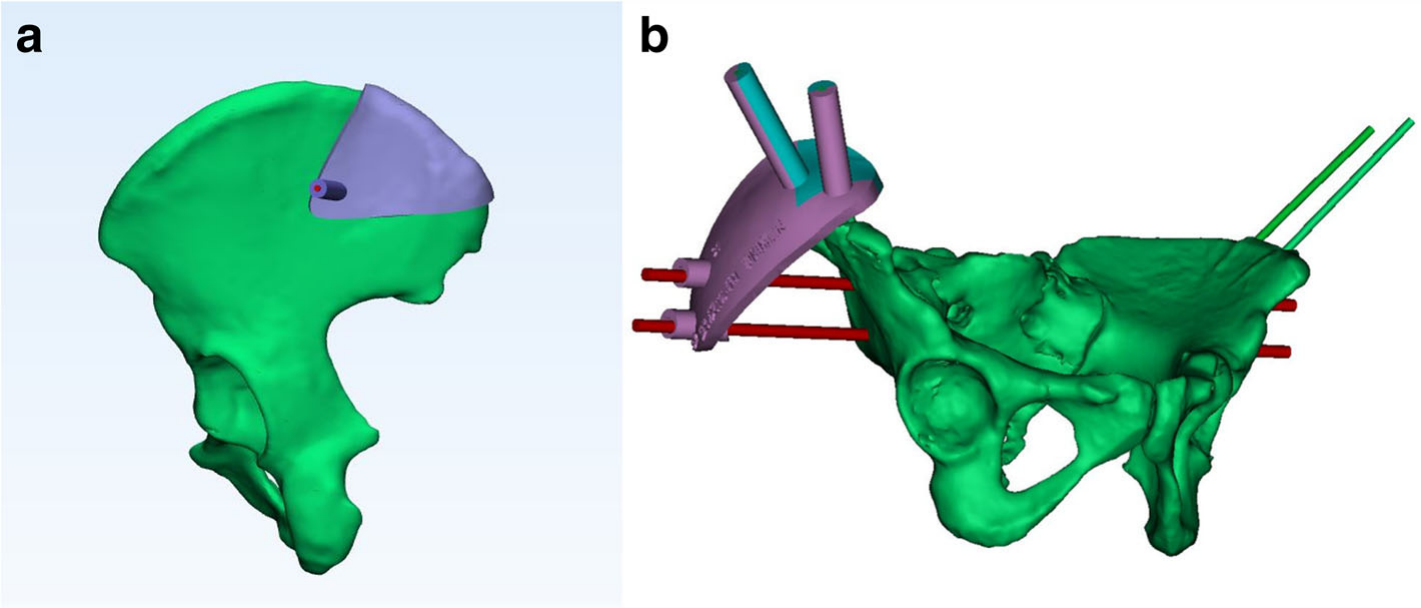
Two types of navigation templates for iliosacral screw insertion. **a** Virtual internal template, docked on the bone surface of the posterior iliac crest. **b** Virtual external template, mounted on the two external fixator pins

Individualized internal templates, based on three-dimensional (3D) image reconstruction and reverse engineering, provide an alternative to computer-guidance for the placement of a K-wire to guide the accurate insertion of the IS screw (Fig. 1a). Although use of an internal template provides an accuracy in screw placement that is similar to that of computer-assisted navigation, it does require an incision of approximately 5 cm and soft tissue dissection to provide a clear bone surface to match the template [8, 9]. Moreover, in our experience, we have found that the geometry of the posterior iliac crest is inadequate to restrain the internal plate, leading to slippage and malposition of the IS screw. As well, soft tissue residues on the bone may also contribute to a mismatch between the template and bone surface, again resulting in malposition of the IS screw.

**Fig. 2:**
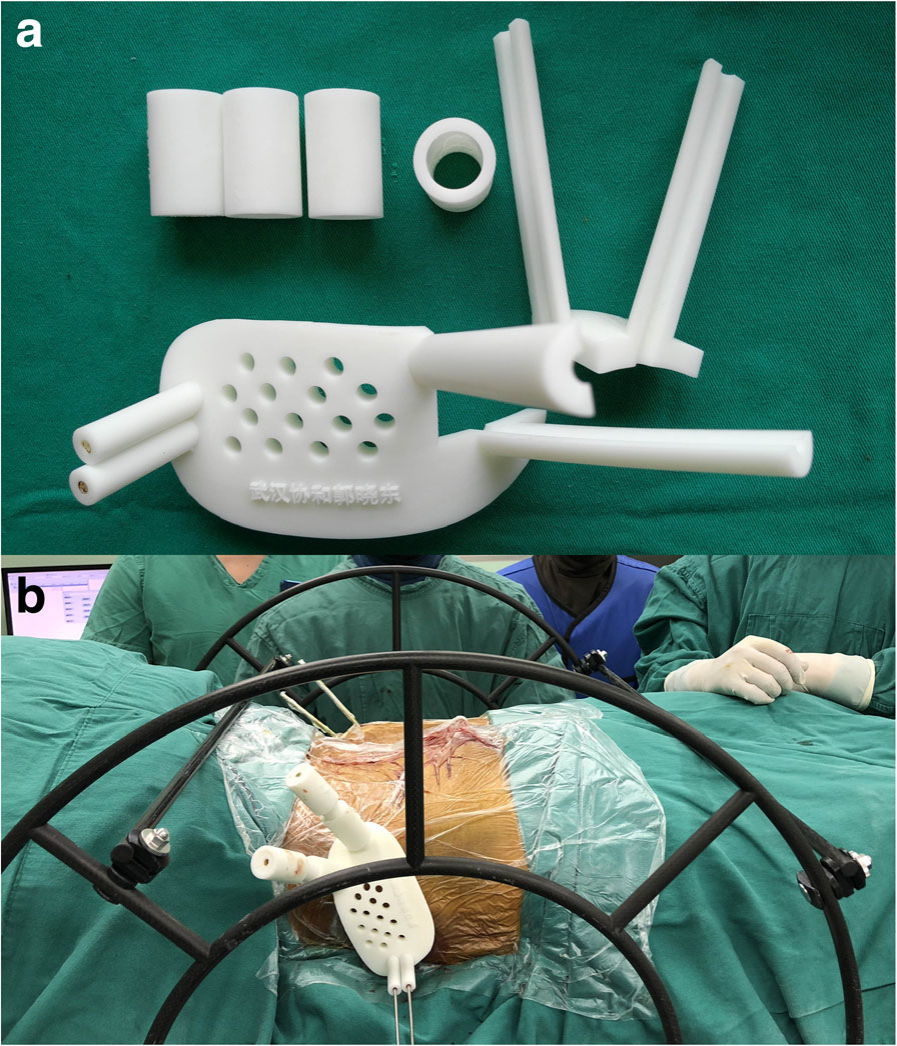
Structure of the external template. **a** The main template contains the mounting and guide sleeves. It was designed to be divided into two parts, through the central axis of the mounting sleeve. **b** Template assembled intra-operatively. The external template can be combined with the pelvic reduction frame as necessary

Emergent external fixation is widely used for the primary management of pelvic fractures. The external fixator pins, which are inserted deep into the iliac crest, could provide a simple geometric surface and solid cornerstone for mounting an external template. Therefore, we developed a low-profile 3-dimensional (3D) printed external template that can be firmly attached to the external fixator pins, overcoming the abovementioned disadvantages of internal templates, to guide IS screw insertion (Fig. 1b and Fig. 2). The aim of our study was to describe the use of our new patient-specific external template to guide the insertion of iliosacral screws and to evaluate the efficacy and safety of this technique compared to the conventional fluoroscopy-guided technique. We hypothesized that use of the 3D-printed external template would lead to a superior outcome, in terms of the accuracy of screw insertion, a shorter operative time and lower radiation exposure, to the conventional technique.

## Methods

### Patients

The study group consisted of 40 patients who were treated at Wuhan Union Hospital, between January 2016 and September 2017, for traumatic incomplete or complete disruptions of the posterior pelvic ring (fractures of types B and C, per the AO/OTA classification) using IS screws [10]. The radiographs and medical records of these patients were retrospectively analyzed (Table 1).

**Table 1:** Demographic and surgery details

	Template group (*n* = 22)	Conventional group (*n* = 18)	*P* value
Sex	Male 11, Female 11	Male 10, Female 8	0.726^*^
Age (years)	51.7 ± 15.2	50.1 ± 13.7	0.728^#^
Time from injury-to-surgery (days)	5.4 ± 2.0	4.1 ± 2.0	0.049^#^
AO/OTA classification of sacrum			0.723^**^
54B	7	4	
54C	15	14	
Posterior pelvic ring disruption, *n* (%)			1.000^**^
SI dislocation	3 (13.6%)	2 (11.1%)	
Denis zone I fracture	8 (36.4%)	7 (38.9%)	
Denis zone II fracture	11 (50.0%)	9 (50.0%)	
Number of screws			0.954^*^
S1	20	16	
S2	18	14	
Operation time (min)			< 0.001^#^
S1	18.7 ± 4.3	39.8 ± 10.6	
S2	17.1 ± 4.7	39.6 ± 11.1	
Average	17.9 ± 4.5	39.7 ± 10.7	
Radiation exposure (cGy/cm^2^)			< 0.001^#^
S1	755.2 ± 239.5	1852.1 ± 844.5	
S2	729.1 ± 226.5	1963.3 ± 872.3	
Average	742.8 ± 230.6	1904.0 ± 844.5	

*P* value < 0.05 considered statistically significant

*Pearson chi-squared test

**Fisher’s exact test

# Two independent samples Student’s t-test

*SI* sacroiliac joint

Values are presented as the mean ± standard deviation, unless otherwise indicated

IS screw placement, using an external template, was performed in 22 patients (template group), including 11 men and 11 women, 51.7 ± 15.2 years of age (range, 18–74 years). All 22 of these patients had undergone external fixation of the pelvic fracture in the emergency department at the time of admission. The conventional fluoroscopy-guided technique of IS screw insertion was used in 18 patients (conventional group), including 10 men and 8 women, 50.1 ± 13.7 years of age (range, 26–75 years). The distribution of the cause and classification of injuries for both groups is summarized in Table 1. All patients had experienced a high-energy trauma, with the distribution for the template and conventional group, respectively, as follows: motor vehicle accident, 9 (40.9%) versus 8 (44.4%); high-energy fall, 6 (27.3%) versus 7 (38.9%); motorcycle accident, 5 (22.7%) versus 3 (16.7%); and machinery injury, 2 (9.1%) versus 0. Using the Denis classification of sacral fractures [11], the distribution of the type of posterior pelvic fractures for the template and conventional group, respectively, was as follows; zone I fractures, 8 (36.4%) versus 7 (38.9%); zone II fractures, 11 (50.0%) versus 9 (50.0%); and sacroiliac joint fractures, 3 (13.6%) versus 3 (11.1%). None of the patients sustained a sacral fracture involving the spinal canal (Denis zone III).

### Marker pin insertion and data collection

Two partially threaded Schanz 5 mm pins (inserted into the iliac crest for emergent fixation at the time of admission) were used as marker pins, as well as provided a stable mounting base for the external template. Consistent with the conventional technique of external fixation of the pelvis, the tips of the two pins were directed in convergent fashion toward the thick bony area above the acetabulum to obtain a firm insertion. Of note, a supra-acetabular pin, which is more commonly used for external fixation of the pelvis than iliac crest screws, was inappropriate for our purpose as it provides inadequate rotational stability to the mounted external template. Moreover, the supra-acetabular pin occupies the supra-acetabular corridor, which is needed to mount one of the pins of the pelvic reduction frame [12, 13] during definitive surgery. In our experience, we found that there was low deformation of the external template itself when it hung perpendicularly from the iliac crest pins (Fig. 2b) in short distance.

Thereafter, all patients in the template group underwent computed tomography (CT) imaging of the pelvis for both trauma assessment and template planning, with additional CT imaging avoided to minimize patients’ radiation exposure. CT data were saved in the Digital Imaging and Communications in Medicine (DICOM) format and used subsequently for design of the template.

The Schanz pins inserted as part of the external fixation of the pelvis were retained in situ until definitive surgery.

### Template design and printing

**Fig. 3:**
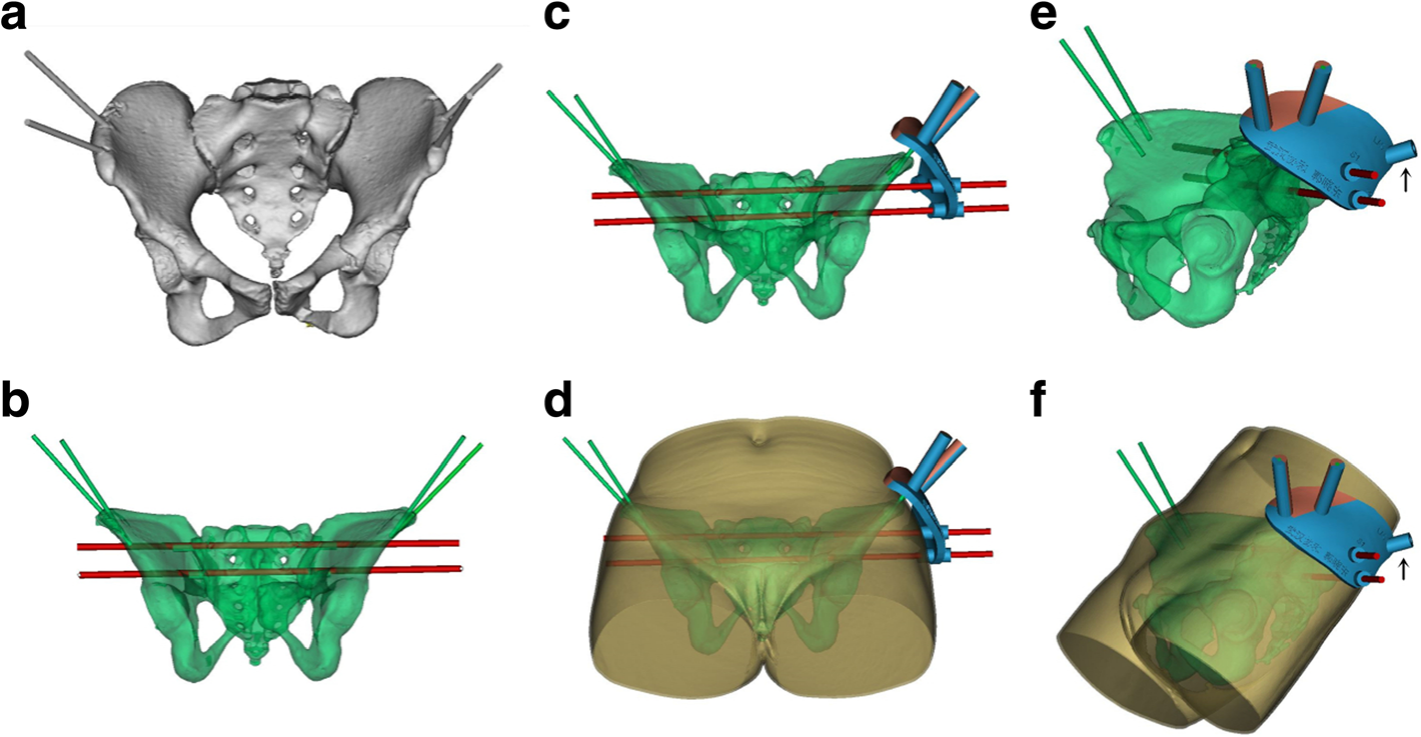
External template designed using Mimics software. **a** A 3D model of the pelvis was reconstructed including the marker pins. **b** S1 and S2 virtual screws were placed into the sacrum and adjusted to the midway of the osseous corridor without any penetration. **c**-**f** The template was designed to connect the marker pins and virtual screws, providing sleeves to attach the template on the marker pins and guide the K-wire to the target corridor. Black arrow indicates the guide sleeve for the anterior column screw. (**d/f**) The plate is low-profile, minimizing the distance between both template and skin, and marker pins and virtual screws

The raw images of the pelvis and skin (DICOM format) were imported into Mimics 10.01 (Materialise, Leuven, Belgium) software for 3D reconstruction. In the 3D model of the pelvis, the trajectory was planned for placement of a virtual screw (7.0 mm diameter) into the S1 or S2 vertebra along the midline of the osseous corridor, with absence of penetration of the cortex confirmed on CT imaging along the three anatomical planes (sagittal, coronal and axial). The placement of another virtual screw into either the injured hemipelvis or contralateral hemipelvis, depending on the type of posterior pelvic ring injury, was also planned. The 3D model (including the bony pelvis, skin and virtual screws) was then exported into an STL format for use with the Geomagic Studio image-processing software (version 12.0; Geomagic, Cary, NC, USA). A virtual template, using the skin model as a substrate, was then designed using the *trim and curve* software, to connect the marker pins and the IS screws. The template design was also exported into the STL format for use with the 3-matic software (Materialise, Leuven, Belgium) to complete the virtual template through Boolean calculation (Fig. 3). The virtual template was then cut into two pieces, through the centric axis of the mounting sleeves so that it could easily be assembled on the marker pins intra-operatively (Fig. 2a). The data from the 3-matic software were imported into the 3D equipment software (UnionTech, SLA-Lite 450 HD, China) to print the external template (accuracy, 0.1 mm; material, photosensitive resin). The components of the template were sterilized, using a low-temperature plasma method, for use intra-operatively.

For patients who did not require fracture reduction, the template was designed to mount on the pins inserted in the iliac crest of the injured hemipelvis, as per the usual method (Fig. 3). For patients with an iliosacral dislocation or sacral fracture (Denis zone I and II fractures), requiring reduction as a component of the definitive surgery, the template was designed to mount on the pins inserted in the contralateral hemipelvis. As the spinal nerves are always contained in the contralateral sacral fragment in Denis zone I and II sacral fractures [14], the template mounted on the contralateral hemipelvis was used for the safe insertion of K-wires, even in cases with poor fracture reduction. However, for external template technique, no ideal solution has been obtained yet for Denis zone III or bilateral sacral fracture needing further reduction.

In our clinical setting, the cost for the design and printing of the template is approximately 250 dollars, requiring about 2 h for the design and more than 10 h for the printing. We cooperate with our affiliated 3D digital orthopaedic center, with a delay between CT imaging and definite surgery of 48–72 h, which takes into account the sterilization process and transportation. This cooperation obviates the need for the hospital to purchase this equipment.

### Surgical technique

**Fig. 4:**
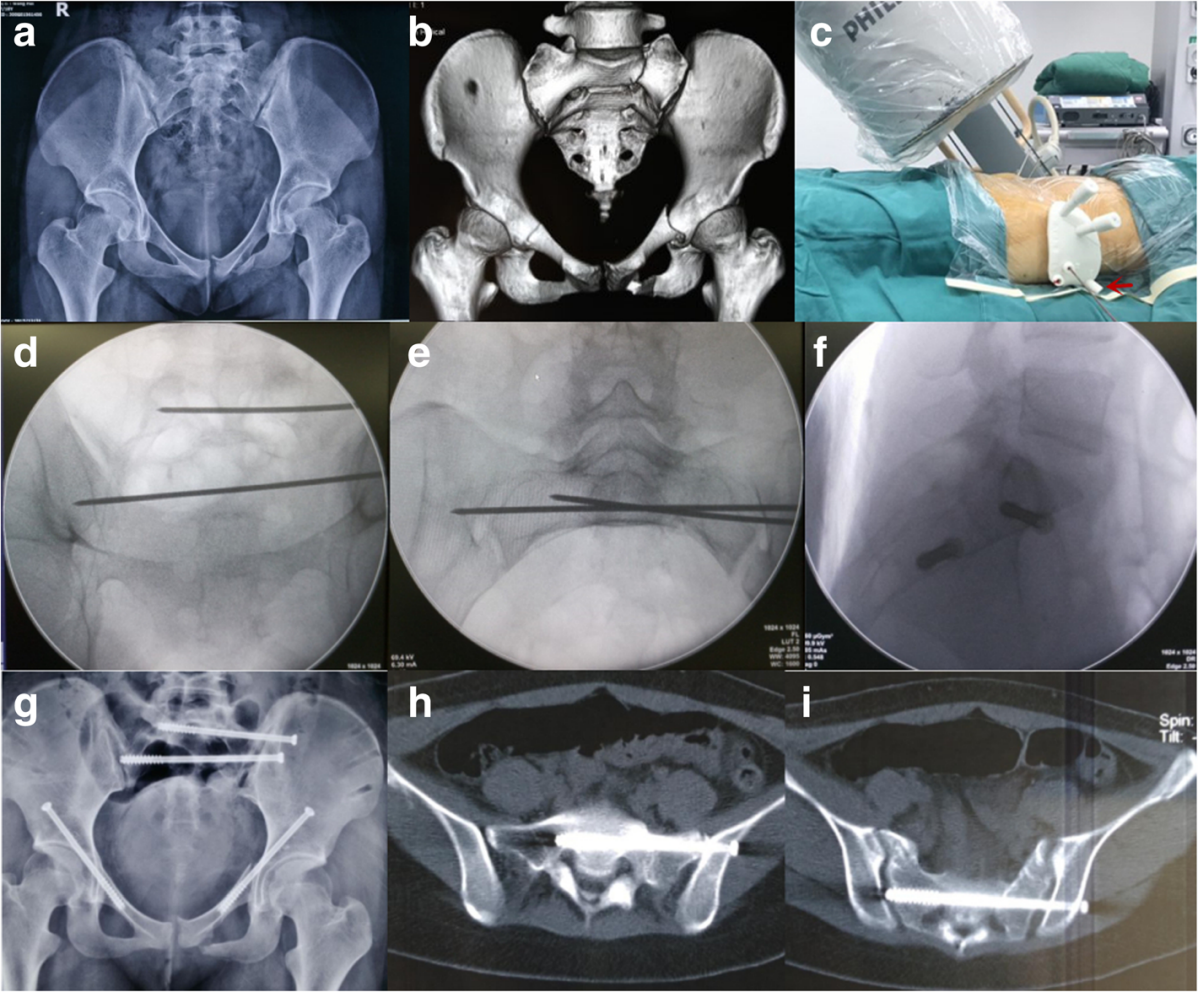
Intraoperative application of the external template. **a-b** Preoperative AP radiographs/3D reconstruction of computed tomography images for a patient with a Denis zone II sacral fracture and bilateral pubic ramus fractures. **c-f** Intraoperative fluoroscopy (**d/e/f** outlet/inlet/lateral view) was used, minimally, to confirm the guide wire and screw in the target corridor. The red arrow in (**c**) indicates the guide sleeve for the anterior column screw. **g-i** Postoperative AP radiograph/computed tomography axial image confirmed the placement of the IS screws

Patients were placed in the supine position on a radiolucent operation table, and surgery was performed under general anesthesia. A fluoroscopic C-arm was placed on the side opposite to the surgeon. The external pelvic fixation frame was removed, retaining the two marker pins to mount the template. The sacral fracture or dislocation was reduced using the pelvic reduction frame described by Lefaivre et al. [12] (Fig. 2b). Then, the external template was mounted on the marker pins and a 2.5-mm K-wire was inserted into the planned corridor using the external template as a reference. Inlet and outlet fluoroscopy views were obtained to confirm the position of the K-wire (with minimum radiation exposure). A 6.5-mm cannulated screw was then inserted, following the K-wire (Fig. 4).

The same procedure was followed for the conventional group, up to the insertion of the K-wire. Specifically, once the sacral fracture or dislocation was reduced, a K-wire was inserted under C-arm fluoroscopy guidance, using lateral, inlet and outlet views. Adjustments in the position of the K-wire under fluoroscopy were made until the correct position was confirmed. A 6.5-mm cannulated screw was then inserted, following the K-wire.

### Measurement and analysis

**Fig. 5:**
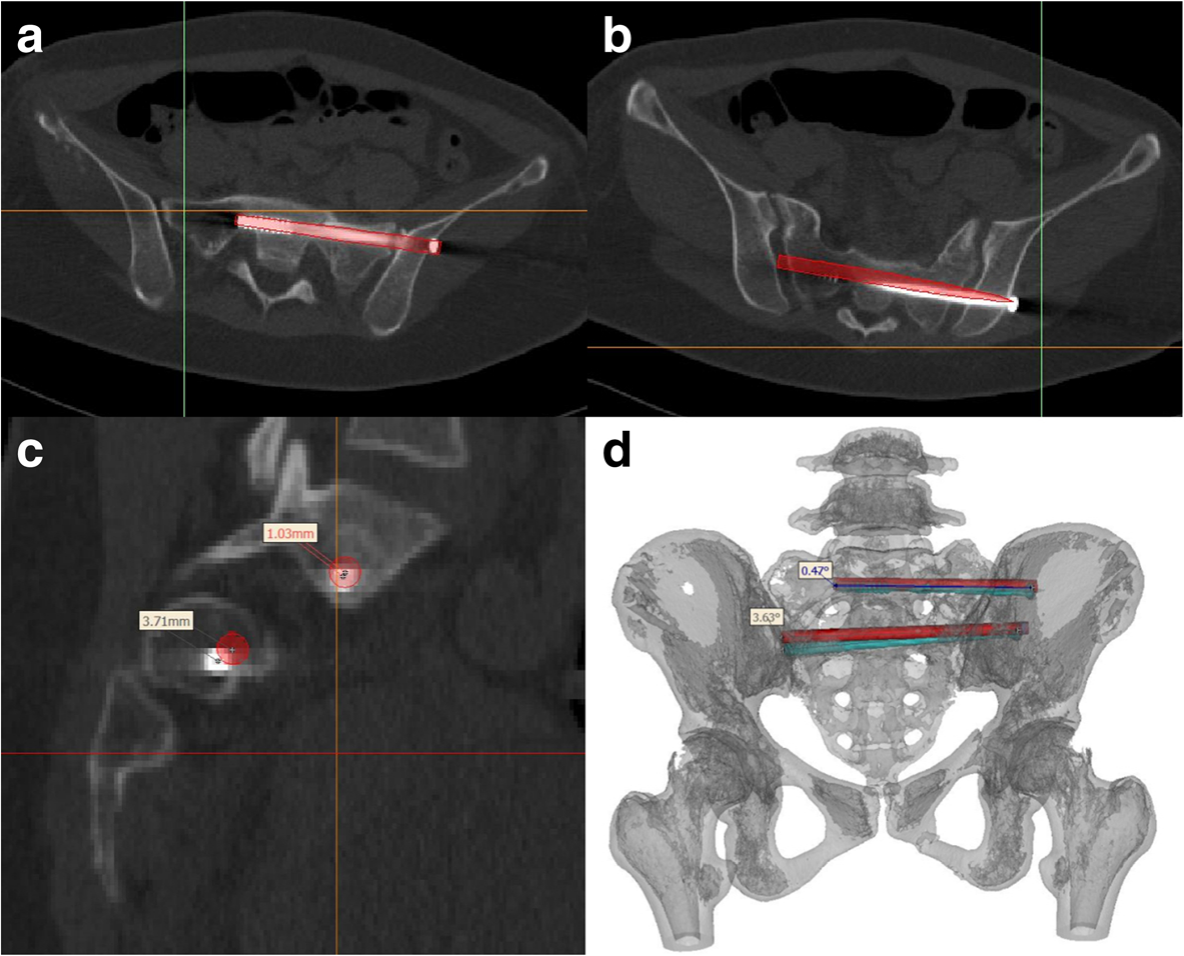
The procedure for postoperative measurements. **a-b** The S1 and S2 axial views obtained after insertion of the partially threaded screws were merged with the pre-operative images used for planning (red bar). **c** The deviation distance between the inserted and planned virtual screw was measured on the sagittal plane at the nerve root tunnel zone. **d** The deviation angle was measured on the superimposed image of the pre- and postoperative 3D reconstructions

Postoperative pelvic radiographs and CT images were reviewed. Two observers independently evaluated the position of the screw from postoperative CT images, using the following grading criteria, as previously described [15]: grade 0, no violation; grade 1, < 2 mm; grade 2, 2–4 mm; and grade 3, > 4 mm. The amount of time required for screw insertion and radiation were also extracted from the surgical records for analysis. The quality of the reduction was graded using the criteria defined by Tornetta and Matta [16]: excellent, ≤4 mm; good, 4–10 mm; and fair, 10–20 mm. By matching pre- and postoperative CT images, the difference between the actual and planned screw positions was quantified by measuring the distance between the two screws at the point of entrance, nerve root tunnel zone and tip, measured in the sagittal plane of reconstructed CT images [17]. The offset angle was also measured (Fig. 5). Quantitative data are presented as the mean ± standard deviation. Between-group differences were evaluated using independent samples Student’s *t*-test, the chi-squared test, and Fisher’s exact test, as appropriate for the data type and distribution. All analyses were performed using SPSS (version 17.0; Chicago, IL, USA).

## Results

A total of 65 screws were implanted in 40 patients: 37 screws (19 S1, 18 S2) in the template group (22 patients) and 28 screws (14 S1, 12 S2) in the conventional group (18 patients). The quality of the reduction was not significantly different between the two groups (Table 2). The average operative time per screw was 17.9 ± 4.5 min (range, 17–52 min) for the template group, which was significantly less than the 39.7 ± 10.7 min (range, 63–115 min) required for the conventional group (*p* < 0.001). The radiation exposure dose was lower for the template (742.8 ± 230.6 cGy/cm^2^) than conventional (1904.0 ± 844.5 cGy/cm^2^) group (*p* < 0.001; Table 1). The rate of screw perforation was also lower in the template (1 of 37 screws, 1 at grade 1) than conventional (4 of 28 screws, 2 at grade 1, 2 at grade 2) group (*P* < 0.001). No incidence of neurovascular injury was identified among cases of screw perforation (Table 3). No incidence of pin site infection was noted among patients treated with emergent external fixation.

**Table 2:** Quality of the reduction

	Excellent (≤4 mm)	Good (4–10 mm)	Fair (10–20 mm)	*P* value
Template group	7	12	3	1.000^*^
Conventional group	5	11	2	

*Fisher’s exact test; *p* < 0.05 considered statistically significant

**Table 3:** The rate of screw perforation

	Grade 0	Grade 1	Grade 2	Perforation	*P* value
Template group	97.4%	2.6%	0%	5.3%	< 0.001*
S1 (*n*)	19	1	0		
S2 (*n*)	18	0	0		
Conventional group	86.7%	6.7%	6.7%	13.7%	
S1 (*n*)	14	1	1		
S2 (*n*)	12	1	1		

*Fisher’s exact test; *p* < 0.05 considered statistically significant

In the template group, the mean distance between the actual and virtual screws was 2.7 ± 0.8 mm (tip), 1.8 ± 0.6 mm (nerve root zone) and 1.5 ± 0.5 mm (entrance point), with a mean deviation angle of 1.8 ± 0.8°.

## Discussion

Use of our novel external template to guide K-wire insertion, and subsequent IS screw placement, provided high accuracy, with a shorter operative time and lower radiation exposure than the conventional fluoroscopy-guided technique. Moreover, despite the conventional use of inlet, outlet and lateral fluoroscopy views to guide percutaneous IS insertion, violation of the cortex remains an inherent problem of IS screw implantation [18–20], with additional fluoroscopic views often required intra-operatively to further improve accuracy. Ozmeric et al. [19] advised that two different inlet views should be used to evaluate the anterior and posterior borders of the sacral body, separately. Kim et al. [18] even suggested using two inlet views (at 25° and 55° from the vertical) and three outlet views (at 25°, 35° and 55° from the vertical) to avoid misperception of the local anatomy. However, any adjustment in the position of the guide wire required in one view should necessarily be reconfirmed on all fluoroscopic views. As such, improving the accuracy of placement of the guide wires using the conventional method increases both intra-operative time and radiation exposure, which limits the use of this iterative approach for accurate guide wire placement in clinical practice. Different assistive tools have been used to improve the accuracy and efficiency in placing guide wires, including the use of multidimensional fluoroscopy [20], an internal template [8, 9], thermoplastic membrane navigation [21], and computer-assisted navigation [5, 6, 17, 22]. Our external template is distinctive from these assistive approaches, with greater pre-operative time taken to design and print the navigation template, which avoids the need for repeated fluoroscopy during the surgery and reduces the overall operative time.

Radiation exposure is a significant concern for percutaneous IS screw insertion. Excessive amounts of radiation can cause muscle weakness, hair loss, cataracts, and even cancer [23]. Computer-assisted navigation does allow a surgeon to exit the operative theatre during image capture, reducing radiation exposure for the surgeon (and medical team). A distinct advantage of our external template is the shorter intra-operative time required to insert the guide wire within the target corridor. As such, the dose of radiation exposure was significantly lower (at 742.8 ± 230.6 cGy/cm^2^), for both patients and the medical staff, compared to the conventional technique (at 1904.0 ± 844.5 cGy/cm^2^).

The accuracy of IS screw insertion has traditionally been evaluated using the penetration grades [15]. In our study, the penetration grade was significantly lower for the external template than conventional group (Table 3). However, due to the ceiling effect, this grading system cannot be used to assess the accuracy of the large number of screws that did not penetrating the bone cortex. Takao et al. [17] described a method to evaluate the deviation distance between the planned and actual IS screw, measured at the tip of the screw, the nerve root tunnel zone and entry point, on sagittal view CT images. Using 3D CT fluoroscopy matching navigation, they reported mean deviations of 2.2 ± 0.8 mm (tip), 1.8 ± 0.7 mm (nerve root tunnel) and 2.5 ± 1.8 mm (entry point). A similar method was adopted by Takeba et al. [22], using the O-arm navigation system, with a mean deviation in the position of the tip of the screw of 1.3 ± 0.6 mm. In our study, we used the Takao et al.’s method, with deviation measures comparable to those of the above studies, in which computer navigation was used to guide IS screw insertion. The satisfactory results we obtained with regard to deviation between the planned and actual screw placement and low rate of penetration into the cortex reflect the precise planning of the IS screw trajectory, which was strictly along the midline of the osseous corridor and was confirmed on axial, coronal and sagittal planes in software. Moreover, the special strategy wherein the template was designed such that it could be mounted on the contralateral hemipelvis helped avoid screw penetration in cases with poor fracture reduction. Furthermore, with experience, we have developed the following tips for designing the template to decrease the extent of deformity of template system. First, the template should have a low profile, which minimizes the distance between the template and the skin and, thus, between the marker pins and the virtual screws (Fig. 3d-e). Second, the distance between the two pins inserted in the iliac crest needs to be sufficiently wide to provide good anti-rotation ability for the external template. Third, the guide sleeve in the external template should closely match the guide wire and be sufficiently long to adequately guide K-wire insertion. Fourth, the template system should be constructed using a high-strength material, such as stainless steel or photosensitive resin, providing a strong restraint to bending.

The delay in definitive surgery because of the time required for the design and printing of the template is a limitation of our technique. The mean time from injury to surgery for the template group in our study was 5.4 days, compared to 4.1 days for the conventional group (*p* = 0.049). This longer delay for the template group, however, did not negatively influence the quality of the fracture reduction, which was comparable between the two groups. Of note, all definitive surgeries of both groups were performed within 14 days of the trauma, and the use of the pelvic reduction frame for closed reduction [13] partly offset any adverse effects of a longer delay to surgery.

Pin site infection, which is a common complication of external fixation [24, 25], is also a potential weakness of our external template technique due to the need to insert pin into the iliac crest. However, there was no incidence of pin site infection in our study group. Use of a strict cleaning protocol for the pin sites [26] and effort to shorten the duration of pin fixation [27] can be important factors in preventing pin site infection. Furthermore, the marker pin insertion is an extra process for patients who did not need emergent external fixation. The decision to use this technique needs careful consideration and informed consent from these patients. The indications for the use of our external template technique among patients who did not require emergent external fixation, include, but are not limited to, the need for insertion of multiple percutaneous screws, dysmorphic sacrum and osteoporotic pelvic fractures.

## Conclusions

In summary, the external template is an effective tool for percutaneous IS screw insertion, decreasing radiation exposure for both surgeon and patients and avoiding neurovascular injury caused by screw malposition. Considering the high accuracy in IS screw placement that we achieved using an external template, we propose that this technique could potentially be used for other percutaneous insertions of screws in pelvic or acetabular surgery (Fig. 3e-f and Fig. 4c). Further applications and studies are still required to confirm this hypothesis.

## Abbreviations

3D: Three-dimensional
CT: Computed tomography
DICOM: Digital imaging and communications in medicine
IS: Iliosacral
